# Delayed onset of severe chronic pain in CASPR2 autoantibody–associated Morvan syndrome in a former UK swine abattoir worker

**DOI:** 10.1097/PR9.0000000000000675

**Published:** 2018-09-17

**Authors:** Andreas Goebel, Austen Peter Moore, Anu Jacob

**Affiliations:** a Pain Research Institute, Faculty of Health and Life Sciences, University of Liverpool, Liverpool, United Kingdom; Departments of b Pain Medicine and; c Neurology, Walton Centre NHS Foundation Trust, Liverpool, United Kingdom

**Keywords:** Neuropathic pain, CASPR2, Morvan syndrome, Abattoir

## Abstract

**Introduction:**

Autoantibody-mediated autoimmunity directed against targets within the voltage-gated potassium channel complex (VGKCC autoantibodies) has been implicated in causing neuropathic pain.

**Methods:**

We report the case of a 76-year-old, United Kingdom male who was diagnosed with contactin-associated protein 2 (CASPR2) autoantibody–associated Morvan syndrome, a rare neurological condition.

**Results:**

He had previously worked in a swine abattoir; exposure to aerosol within swine abattoirs has been reported to elicit an immune response resulting in the production of these autoantibodies; however, unusually, his manifestations emerged with several years' latency. Although this patient's Morvan syndrome–associated seizures were well-controlled with antiepileptic drugs, his neuropathic pain and painful muscle fasciculations did not respond to pharmacological interventions. He refused pain management program treatment, but high-dose immunoglobulin treatment or treatment with rituximab, reported to be sometimes effective in this group, was not initiated because of concerns regarding his general frailty.

**Discussion and Conclusion:**

This case highlights issues around the identification and treatment of rare patients with chronic pain who have voltage-gated potassium channel complex autoantibodies; it also emphasizes the possibility that former swine abattoir workers might be at risk of developing neuropathic pain even years after their vocational exposure.

## Abstract 2

Supplemental Digital Content is Available in the Text.

## 1. Introduction

A 76-year-old gentleman attended the outpatient clinic at our Department of Pain Medicine in 2016 after referral by his neurologist. He complained of severe pain with a toothache-like character on the back of both legs, extending from the buttocks to the knees; in addition, he had recent onset severe burning pain affecting the outside of the left knee and the right groin.

Review of his medical notes revealed variability of his complaints. In previous consultations, by other departments, he had complained of “firing, cramping, spasmodic pain,” occurring in intervals of several days and affecting medial–posterior thighs, and feet below ankles.

His medical history is given in the supplementary appendix A (available at http://links.lww.com/PR9/A28). He had first developed nonpainful, muscle-related problems 5 years before presentation to us, with episodes of sudden involuntary twitching of eyelid muscles (myokymia) and fasciculating facial muscle movements. Initial electromyography showed myokymic discharges in orbicularis oculi bilaterally, but not abnormalities in limb muscles. Several months later, he had been observed to have “vacant spells.” On assessment by a neurologist, routine blood tests, computed tomography, and magnetic resonance imaging of the brain did not show significant abnormality. An electroencephalogram showed focal epileptiform discharges in the right midtemporal area, with simultaneous bradycardia. He was diagnosed as having complex partial seizures and started on sodium valproate with good improvement of his vacant episodes.

Four months later (in 2012), he had started to notice prominent cramps and twitches in his legs, ankles, and thighs, in addition to a constant “tight uncomfortable feeling almost amounting to pain”, and then shortly thereafter, he first reported pain in his back and thighs. He did not complain about dysautonomia, and his sleep was only intermittently disturbed.

He was noted as thinly built. There was wasting of calf and thigh muscles with muscle fasciculations. The rest of his neuroexamination was normal, so that at this point, no clear cause for his pain was found. He underwent further investigations. Repeat magnetic resonance imaging whole spine and nerve conduction tests were normal. Electromyography showed myokymic discharges in both orbicularis oculi, and scanty but widespread fasciculation in multiple large leg muscles, in the absence of denervation. Careful electromyography analysis on 2 occasions revealed no evidence for neuromyotonia or myokymic discharges in limb muscles. Standard autoantibody tests and antiglutamic acid decarboxylase or anti *N*-methyl-d-aspartate receptor antibodies were negative. However, the serum voltage-gated potassium channel complex (VGKCC) antibody titer was elevated and remained raised on repeated testing. Detailed testing with cell-based assays (John Radcliffe Hospital Oxford) showed elevated CASPR2 (contactin-associated protein-like 2) antibodies. CASPR2 antibody levels rose over 2 to 3 years from 250 to 700 pmol/L (positive >125) and then remained stable. CASPR2 antibodies can be paraneoplastic,^10^ but additional extensive investigations returned no abnormal results (supplementary appendix B, available at http://links.lww.com/PR9/A28).

The most likely neurological diagnosis was deemed to be VGKCC (CASPR2)-associated peripheral (peripheral nerve excitability and pain, weight loss), and central (epilepsy) syndrome—Morvan syndrome,^5^ with no identified underlying trigger. Because his seizures were well-controlled with valproate, the reported most distressing symptoms were pains of mixed character and muscle fasciculations/cramps.

The patient's pain was diagnosed as neuropathic by his neurologist in 2014. At that time, he was noted to recently have received steroids for treatment of an exacerbation of his concomitant chronic obstructive pulmonary disease (60-mg prednisolone × 3 days) and experienced moderate pain relief. A pragmatic trial as an inpatient on 40-mg prednisolone daily for several weeks, specifically to reduce his distressing and painful symptoms was unsuccessful. He was tried on several analgesic medications without benefit, including tramadol, pregabalin to maximal dose, amitriptyline, and duloxetine. Buprenorphine patches afforded moderate, short-lived pain reduction, but he developed respiratory problems, with consequent admission to A&E, and this had to be stopped. Low-dose oxycontin/naloxone at 5 mg/2.5 μg was ineffective, and dose increase caused inacceptable side effects, including reduced cognition. There was short-lived pain reduction to dihydrocodeine, whereas tapentadol at a low dose (50-mg BD) was ineffective. Although his pain intensity a year before he was seen by us was documented as only moderate, with 5/10 on an 11-point scale (0 = no pain, 10 = pain as bad as you can imagine), the clinical impression by his treating team was of a highly distressing problem.

During assessment at our Department of Pain Medicine in 2016, the patient revealed that he had worked as a swine abattoir worker for many years up to the age of 60 years (7 years before his first presentation with abnormal muscle symptoms). He had then worked as a butcher a further 5 years until age 65 when he retired.

Because VGKCC antibody–associated neuropathic pain has been reported responsive to disease modification with immune-modulating drugs,^6^ immune interventions were now further discussed but were found unsuitable because of this patient's frailty and the lack of sufficient evidence. He was offered attendance at a pain management program. He was first keen to attend but later indicated that he was instead looking for a pain cure. His mixed pains mainly affecting his legs were ongoing at the end of 2017.

## 2. Discussion

We report an unusual case of delayed-onset intrusive chronic neuropathic pain in a former swine abattoir worker diagnosed with CASPR2 autoantibody–associated Morvan syndrome. To our knowledge, this is the first published case of a swine abattoir worker presenting with painful signs and symptoms (1) outside the United States, (2) with a latency period of several years after vocational exposure, (3) with Morvan syndrome.

**Figure 1.**
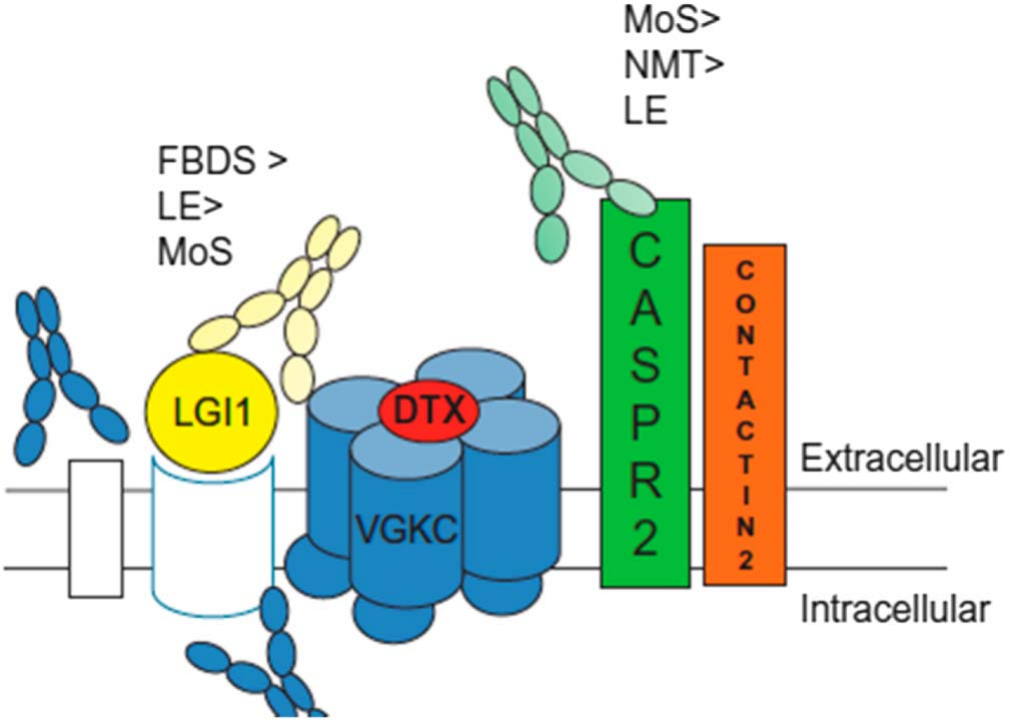
The VGKCC and associated proteins with LGI1- and CASPR2-binding autoantibodies and associated neurological conditions. DTX, dendrotoxin known to directly bind VGKCC; FBDS, faciobrachial dystonic seizures; LE, limbic encephalitis; LGI1, leucine-rich glioma-inactivated 1; MoS, Morvan syndrome; NMT, neuromyotonia; VGKCC, voltage-gated potassium channel complex. Note some patients that are negative to both LGI1 and CASPR2 may have antibodies binding the intracellular domain of this protein complex, but their pathophysiological role is uncertain. Recent evidence suggests that painful neuropathic symptoms can arise in both CASPR2-positive and LGI1-positive patients (from Irani SR, Vincent A. Handbook of Clinical Neurology 2016,^5^ with permission).

For practitioners in pain medicine, this case highlights the emerging field of chronic pain associated with autoantibody-mediated disorders^4^; it also emphasizes complexities around potassium channel complex autoantibody–associated chronic pains (Fig. 1). Antibodies to the VGKCC were initially identified by radioimmunoassay based on dendrotoxin, which binds to shaker-type voltage-gated potassium channel antibodies. Later experiments showed, however, that the antibodies do not directly bind to the potassium channels but to proteins with which they complex, including CASPR2 and LGI1.^5^ Generally, although potassium channels act as “brakes” for sensory neuron excitability, the autoantibodies that bind to CASPR2 reduce surface expression of potassium channels and consequently cause hyperexcitability.^2^

(1) Potassium channel autoantibody complex (VGKCC)-associated pain is typically reported as a feature in rare neurological disorders caused by these antibodies, such as neuromyotonia or myokymia (Morvan syndrome); these disorders can sometimes be paraneoplastic.^1,2,5^
(2) A separate group of patients have neuropathic pain and autoantibodies to CASPR 2, without having any neurological abnormalities; encouraging results on pain relief after treatment with immune therapies have been communicated.^6^
(3) A third group of patients with VGKCC-associated pain was described in 2010/2012 by Mayo clinic neurologists. These patients were swine abattoir workers, who developed acute or subacute sensory-predominant polyradiculoneuropathy.^7,9^ Workers closest to the swine brain processing machinery were most affected, and it was believed that patients had effectively been immunized by the inhalation of aerosolized swine brain antigens, to now produce autoantibodies directed against their own peripheral nerves. Brain manifestations were rare, and no patient had Morvan syndrome. Their pain was frequently aching and burning, involving mostly legs (86%), arms, and the head/neck region. The autoantibody serum titers in these patients reduced over time, either spontaneously with the stop of exposure to swine brain aerosol or with immune modulation treatment.

Our patient's CASPR2-positive, painful neurological condition, and his previous work as swine abattoir worker may be coincidental. However, given the rarity of each, we suggest this is unlikely, and it is more likely that a past exposure to swine neural tissue aerosol caused both his central neurological signs that were brought under control, and the peripheral neuropathic pain for which we found no effective treatment. We speculate that he might have had low-level CASPR 2 antibodies for several years before symptoms started.

A limitation of our study was that we did not examine the patient for additional neuropathic pain diagnoses such as small fiber neuropathy.

There are diagnostic and therapeutic challenges concerning CASPR2-associated chronic pain in general and abattoir-working in specific:

(1) Although in this case the central and peripheral neurological signs and symptoms were a “give away,” how can we clinically be alerted to the possibility that a particular patient's peripheral neuropathic pain may be caused by CASPR2 autoantibodies?
(2) Although there is some evidence for the efficacy of immune treatments,^3,6,8^ given the lack of randomized controlled trials, and the rarity of this condition, how shall we consider immune therapy to reduce pain in either case, CASPR2 seropositivity with/without neurological disorders?
(3) Should former swine abattoir workers be screened for neuropathic pain and possibly as late as 5 to 10 years after stop of their exposure?

In summary, we present a case of a patient with peripheral neuropathic pain, CASPR2 serum autoantibodies, and Morvan syndrome caused very likely by exposure to swine neural-tissue aerosol several years ago. This case also highlights that availability of internationally agreed guidance on diagnosis and treatment of VGKCC autoantibody–associated neuropathic pain would be useful to support clinical practice in pain medicine.

## Disclosures

The authors declare no conflict of interest with regards to this study.

This study was funded by internal sources.

## Appendix A. Supplemental digital content

Supplemental digital content associated with this article can be found online at http://links.lww.com/PR9/A28.
